# Organotypic vibrosections from whole brain adult Alzheimer mice (overexpressing amyloid-precursor-protein with the Swedish-Dutch-Iowa mutations) as a model to study clearance of beta-amyloid plaques

**DOI:** 10.3389/fnagi.2015.00047

**Published:** 2015-04-09

**Authors:** Christian Humpel

**Affiliations:** Laboratory of Psychiatry and Experimental Alzheimer's Research, Department of Psychiatry and Psychotherapy, Medical University of InnsbruckInnsbruck, Austria

**Keywords:** Alzheimer, beta-amyloid plaques, clearance, *in vitro* model, organotypic brain slices, vibrosections

## Abstract

Alzheimer's disease is a severe neurodegenerative disorder of the brain, pathologically characterized by extracellular beta-amyloid plaques, intraneuronal Tau inclusions, inflammation, reactive glial cells, vascular pathology and neuronal cell death. The degradation and clearance of beta-amyloid plaques is an interesting therapeutic approach, and the proteases neprilysin (NEP), insulysin and matrix metalloproteinases (MMP) are of particular interest. The aim of this project was to establish and characterize a simple *in vitro* model to study the degrading effects of these proteases. Organoytpic brain vibrosections (120 μm thick) were sectioned from adult (9 month old) wildtype and transgenic mice (expressing amyloid precursor protein (APP) harboring the Swedish K670N/M671L, Dutch E693Q, and Iowa D694N mutations; APP_SDI) and cultured for 2 weeks. Plaques were stained by immunohistochemistry for beta-amyloid and Thioflavin S. Our data show that plaques were evident in 2 week old cultures from 9 month old transgenic mice. These plaques were surrounded by reactive GFAP+ astroglia and Iba1+ microglia. Incubation of fresh slices for 2 weeks with 1–0.1–0.01 μg/ml of NEP, insulysin, MMP-2, or MMP-9 showed that NEP, insulysin, and MMP-9 markedly degraded beta-amyloid plaques but only at the highest concentration. Our data provide for the first time a potent and powerful living brain vibrosection model containing a high number of plaques, which allows to rapidly and simply study the degradation and clearance of beta-amyloid plaques *in vitro*.

## Introduction

Sporadic Alzheimer's disease (AD) is a progressive chronic neurodegenerative disorder (at least 97% of all cases are non-genetic) and is characterized by severe beta-amyloid (Aβ) deposition in brain (plaques) and in vessels (cerebral Aβ angiopathy, CAA), Tau pathology, cell death of cholinergic neurons, microglial activation, inflammation and cerebrovascular damage. The causes of AD are yet unknown, but so far the Aβ cascade (Selkoe, 2002) is the most prominent hypothesis and is thought to be the primary event that triggers the pathological cascade in AD. However, there is still an ongoing controversy if Aβ plaques directly cause the disease, or if Aβ plaques are generated secondary to another (e.g., vascular) pathological event.

The amyloid precursor protein (APP) is cleaved by secretases into Aβ peptides (40, 42, or 43 amino acids), and these peptides aggregate under certain conditions and are deposited as Aβ plaques. The plaques consist of a central core of highly aggregated Aβ peptides and a halo around the plaques (Wisniewski et al., 1989). It is well established that the “halo” consists of degenerating nerve fibers and several infiltrating cells, like reactive astrocytes or microglia. The most robust deficits occur in this halo around the plaques within a radius of approximately 20 μm, including local alterations in spine density, neuritic curvature, calcium dysregulation and oxidative stress (Xie et al., 2013). The “core” consists of a dense aggregation of large or several small Aβ depositions, especially the toxic Aβ_1–42_. Brain capillary vessels penetrate the core and it seems that every plaque is associated with a vessel.

In severe AD, the whole brain is filled with several Aβ plaques. Therapeutic options aim to prevent the deposition of plaques (beta-sheet breaker, secretase inhibitors, Aβ-degrading enzymes) to degrade and clear the plaques (Glabe, 2000; Wang et al., 2006). Immune cells, especially microglia are of special interest, because they (or transformed macrophages) may phagocyte plaques. However, in AD the phagocytic activity of these microglial cells is diminished (D'Andrea et al., 2004; Lee and Landreth, 2010). Thus, strategies explore if endogenous blood cells, especially monocytes, may enter the brain and migrate to plaques; indeed in the AD mouse model this has been demonstrated (Lebson et al., 2010; Hohsfield et al., 2014). Alternatively, it seems likely that infusion of plaque-degrading enzymes may be an attractive strategy to destruct plaques. Indeed, several Aβ-degrading proteases have been identified, including neprilysin (NEP; Iwata et al., 2001; Leissring et al., 2003; Marr et al., 2003), insulin-degrading enzyme (IDE; Qiu et al., 1998; Bennett et al., 2000; Kurochkin, 2001; Farris et al., 2003; Leissring et al., 2003), endothelin-converting enzyme (ECE; Eckman et al., 2001, 2002), angiotensin-converting enzyme (ACE; Hu et al., 2001), plasminogen activators (Van Nostrand and Porter, 1999; Ledesma et al., 2000; Tucker et al., 2000), myelin basic protein (Liao et al., 2009) or different matrix metalloproteinases, such as e.g., MMP-2 (gelatinase A/type IV collagenase) (Roher et al., 1994; Yamada et al., 1995) and MMP-9 (Backstrom et al., 1996; Yin et al., 2006).

However, these proteases have partly a wide range of substrates, are large molecules, and cannot pass the bood-brain barrier (BBB) and find their targets. Different strategies have been tested, especially *in vivo* in AD mouse models. However, these animal models are all very complex, either because the proteases need to be overexpressed using adeno- or lentiviral vectors (Guan et al., 2009; Liu et al., 2010) or because the enzymes are intracranially injected directly into the brain (Park et al., 2013; Walker et al., 2013) or peripherally applied (e.g., i.v.). Further, studies failed due to the lack of BBB penetration or the short half-life of the enzymes (Davis et al., 2004; Henderson et al., 2014). Thus in order to test a time- and dose specificity and selectivity and a possible combination of these enzymes, we certainly need easy to use *in vitro* models.

The aim of the present study was to develop a simple *in vitro* model which contains plaques and can be used to screen for plaque-degenerating enzymatic activity. So far no *in vitro* models exist, which contain plaques. As we have long-time experience in organotypic brain slices, we aimed to develop an *in vitro* organotypic vibrosection model which contains plaques and can be used to screen for clearance activities. We report here for the first time, that vibrosections prepared from adult whole brain Alzheimer mice are useful to screen different Aβ-degrading enzymes and this method is easy, fast and potent.

## Materials and methods

### APP_SDI mice and controls

Wildtype (C57BL/6N) and transgenic APP_SDI (expressing amyloid precursor protein (APP) harboring the Swedish K670N/M671L, Dutch E693Q, and Iowa D694N mutations; C57BL/6-Tg(Thy1-APPSwDutIowa)BWevn/Mmjax) mice were purchased from The Jackson Laboratory and housed at the Medical University of Innsbruck animal facility providing open access to food and water under 12/12 h light-dark cycles. The mice have been generated and extensively characterized by Davis et al. (2004).

### Organotypic vibrosections of adult mice

For vibrosection cultures 9 month old adult mice were used. All experiments conformed to Austrian guidelines on the ethical use of animals and all efforts were made to minimize the number of animals used and their suffering. For all experiments a minimum of *n* = 6 slices was analyzed. Usually 2 slices per group including a control were incubated, which was repeated at least in 3 independent experiments. Vibrosections were performed as described in detail under sterile conditions (Ullrich et al., 2011). The animals were rapidly sacrificed, the brains dissected and sagittally cut. The brains were glued (Glue Loctite) onto the chuck of a water cooled vibratome Leica VT1000A (UV sterilized and under a Laminar Flow), and triggered close to a commercial shave racer. Under aseptic conditions, 120 μm vibrosections were cut and collected in sterile medium. The organotypic vibrosections were carefully placed onto a sterile 0.4 μm pore membrane (Millipore HTTP02500), which was then placed into a 0.4 μm membrane insert (Millipore PICM03050) within a 6-well plate. Vibrosections were cultured in 6-well plates (Greiner) at 37°C and 5% CO_2_ with 1.2 ml/well of the following culture medium: 50% MEM/HEPES (Gibco), 25% heat inactivated horse serum (Gibco/Lifetech, Austria), 25% Hanks' solution (Gibco), 2 mM NaHCO3 (Merck, Austria), 6.5 mg/ml glucose (Merck, Germany), 2 mM glutamine (Merck, Germany), pH 7.2. Vibrosections were incubated for 2 weeks where they attach to the membranes. In order to study if slices develop plaques, vibrosections from 4 month old APP_SDI Tg mice (which display no plaques at this stage) were incubated for up to 5 months.

Recombinant proteases were dissolved in 100 μl sterile medium, aliquoted and stored frozen at −20°C; at the day of use, aliquots were diluted in sterile medium and not filtered. Vibrosections were cultured with or without 1, 0.1, or 0.01 μg/ml recombinant human neprilysin/CD10 (R&D, Nr. 1182-ZNC-010), human insulysin/IDE (R&D systems, nr. 2496-ZN-010), human matrix-metalloproteinase-2 (MMP-2; Peprotech Nr. 420-02) or human matrix-metalloproteinase-9 (Sigma, nr. M8945). At the end of the experiment, vibrosections were fixed for 3 h at 4°C in 4% paraformaldehyde (PAF)/10 mM phosphate-buffered saline (PBS) and then stored at 4°C in PBS until use.

### Immunohistochemistry

Immunohistochemistry was performed as previously described under free-floating conditions (Ullrich et al., 2011). The vibrosections were washed with PBS and incubated in PBS/0.1% Triton (T-PBS) for 30 min at 20°C while shaking. To quench endogenous peroxidase, sections were treated with PBS/1%H_2_O_2_/5% methanol. After incubation, the sections were then blocked in T-PBS/20% horse serum (GIBCO Invitrogen)/0.2% BSA (SERVA) for 30 min at 20°C shaking. Following blocking with mouse IgG blocking reagent (Vector MKB-2213), brain sections were incubated with primary antibody [beta-amyloid, 1–16 (6E10) Covance SIG-39300; glial fibriallary acid protein GFAP Millipore AB5541; microglial Iba1 Wako 019-19741] in T-PBS/0.2% BSA overnight at 20°C. The sections were then washed and incubated with the corresponding biotinylated secondary (Aβ-mouse; GFAP-chicken; Iba1-rabbit) antibody (1:200, Vector Laboratories) in T-PBS/0.2% BSA for 1 h at 20°C shaking. Following secondary antibody incubation, sections were rinsed with PBS and incubated in avidin-biotin complex solution (Elite ABC kit, Vector Laboratories) for 1 h at 20°C shaking. Finally, the sections were washed with 50 mM Tris-buffered saline (TBS) and then incubated in 0.5 mg/ml 3,3′-diaminobenzidine (DAB, Sigma)/TBS/0.003% H_2_O_2_ at 20°C in the dark until a signal was detected. Once DAB staining was visible, the reaction was stopped by adding TBS to the sections. The brain sections were rinsed with TBS, inserted into a 6-well plate (slice down to well surface) directly onto a drop of Vectashield (Vector), cover-slipped and then evaluated under an inverse microscope (Leica DM IRB). Some sections were stained with Thioflavin S (Sigma) to label plaques. Some sections were stained for fluorescence using Alexa-488 (Aβ) and using Alexa-546 (GFAP).

### Western blot

Western blot analysis was performed as previously described by us (Hohsfield et al., 2014). Slices were incubated for 2 weeks, and all slices from 3 wells were taken and pooled in an Eppendorf tube, then dissolved in 100 μl ice-cold PBS containing a protease inhibitor cocktail (P-8340, Sigma), homogenized using an ultrasonic device (Hielscher Ultrasonic Processor, Germany) and then centrifuged at 16,000 × g for 60 min at 4°C. The supernatant was collected (=soluble extract) and the pellet was dissolved in 50 μl 70% formic acid, vortexed and neutralized with 150 μl 7N NaOH (=insoluble extract). Then, 20 μl of the extracts were loaded onto 10% Bis-Tris SDS-polyacrylamide gels, and separated for 25 min at 200 V and then electrotransferred to nylon-PVDF Immobilon-P*^SQ^* membranes for 90 min at 30 V in 20% methanol blotting buffer. The Western Breeze Chromogenic System was used for the detection of specific proteins in cortical extracts. Briefly, blots were blocked for 30 min in blocking buffer, incubated with primary antibodies against beta-amyloid (1:1000) at 4°C overnight, washed, and then incubated in alkaline phosphatase conjugated anti-mouse IgG for 30 min. After washing, bound antibodies were detected using an enhanced chemiluminescence (ECL) system. As a control aggregated beta-amyloid standards (Calbiochem Aβ_1–42_, PP69, Mw = 4417 kDa) were loaded. Aggregation was performed as described by Ryan et al. (2010). Briefly, 250 μg recombinant Aβ_1–42_was dissolved in 250 μl 5% acidic acid (226 μM), 10 min sonicated in a water bath, then diluted to 100 μM in PBS + 0.005% sodiumdodecylsulfate (SDS), then incubated overnight at 4°C, and further diluted in PBS to 11 μM, and incubated for 2 weeks at 4°C. Then the aggregates were further incubated for 2 weeks at 37°C with or without 1 μg/ml MMP-9.

### Evaluation of plaques

Vibrosections at the cortical level were photographed with the Leica inverse microscope at a 10× magnification under a red filter. The exposure time was always 23 ms with the bright light set at the lowest level. The software Openlab was used at a Mac computer connected to the microscope. Pictures were saved as JPG files and the analysis was performed using Image J. The pictures were transformed to a 8-bit grayscale image. The calibration was set at 0.470 (distance in pixels), 1.00 (known distance), 1.0 (pixel aspect ratio) and μm (unit in lenght) and global was activated. The picture was transformed into a binary image and the threshold was adapted to 30–40. The number of particles was counted setting the size to 100–8000 μm^2^. The number of plaques was counted in a defined circle in 2.5 mm^2^ area. Examples of plaque evaluations are given in the **Figures 4E–G**.

### Statistical analysis

Statistical analysis was performed by a Kruskal–Wallis test and Dunn's *post-hoc* test, where *p* < 0.05 represents significance.

## Results

### Plaques in the 9 month old APP_SDI mice

**Figure 1:**
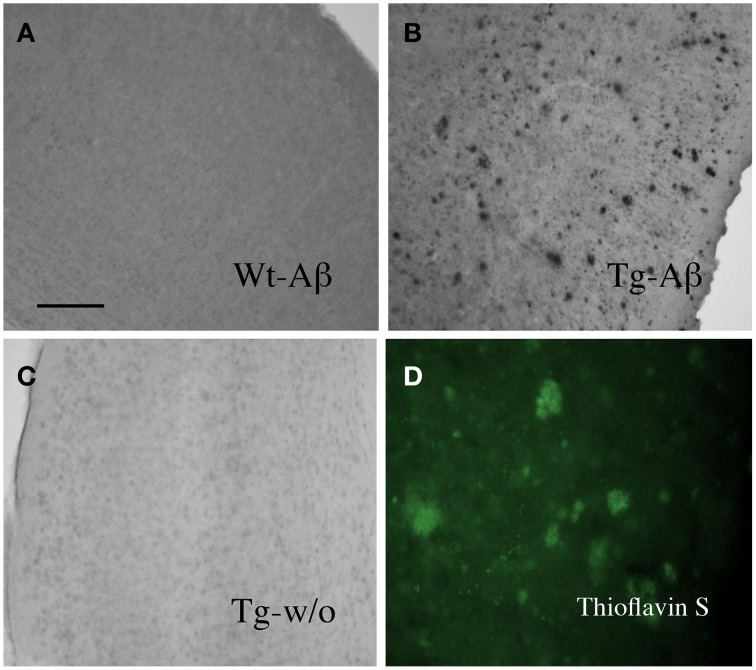
**Beta-amyloid plaques in the amyloid-precursor protein_SDI mouse model vibrosections**. Organotypic vibrosections (120 μm thick) of 9 month old wildtype (Wt, **A**) or transgenic (Tg, **B–D**) mice were prepared and cultured for 2 weeks, then fixed and immunohistochemically stained for beta-amyloid **(A–C)** or Thioflavin S **(D)**. As a control Tg sections were stained without primary antibody **(C)**. Note a dense number of plaques in 2 week old cultured vibrosections of 9 month old APP_SDI mice **(B)**. Scale bar in *A* = 200 μm **(A–C)** and 120 μm **(D)**.

**Figure 2:**
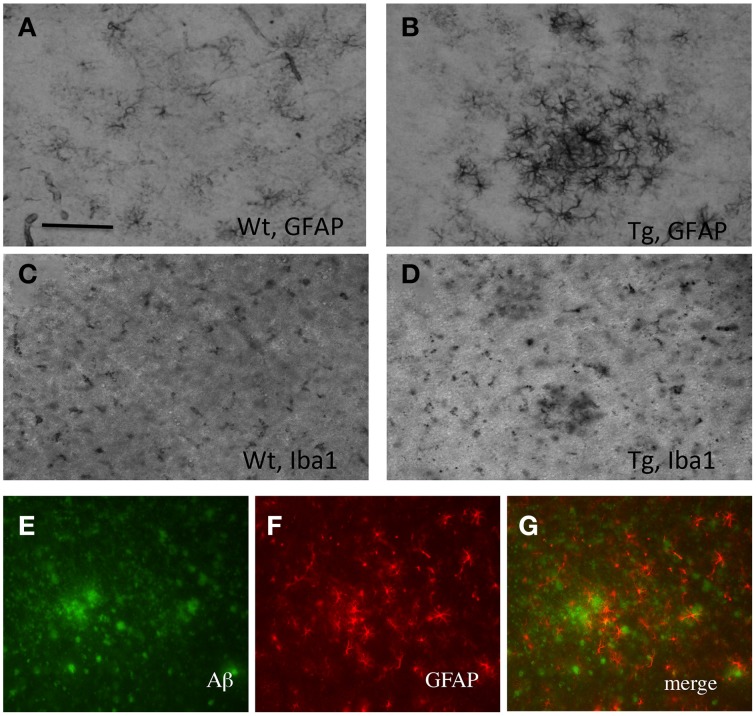
**Characterization of plaques in vibrosections of 9 month old amyloid-precursor protein_SDI transgenic mouse model**. Organotypic vibrosections of 9 month old wildtype (Wt; **A,C**) or transgenic (Tg; **B,D–G**) mice were prepared (120 μm thick) and cultured for 2 weeks, then fixed and immunohistochemically stained for astroglial glial-fibrillary acidic protein (GFAP; **A,B,F**) or microglial Iba1 **(C,D)**. Astroglial GFAP **(F)** was co-stained with beta-amyloid **(E)**, showing reactive astroglia around plaques **(G)**. Scale bar in *A* = 150 μm **(A–D)** and 100 μm **(E–G)**.

Organotypic vibrosections were cultured for 2 weeks and the plaques were stained by immunohistochemistry for Aβ or Thioflavin S. In cultured wildtype mice vibrosections (9 month old, 120 μm, 2 weeks) no plaques were visible (Figure 1A). In cultured APP_SDI mice vibrosections a high number of plaques were stained by Aβ immunostaining (Figure 1B) or Thioflavin S dye (Figure 1D). As a control the primary antibody was omitted in transgenic vibrosections and no plaques were found (Figure 1C). It became evident, that reactive astrocytes expressing GFAP were markedly enhanced in transgenic (Figure 2B) but not wildtype (Figure 2A) vibrosections, which centered around the Aβ plaques (Figures 2E–G). Similarly, microglial Iba1 staining was markedly increased in transgenic versus wildtype vibrosections (Figures 2C,D).

### Development of plaques

**Figure 3:**
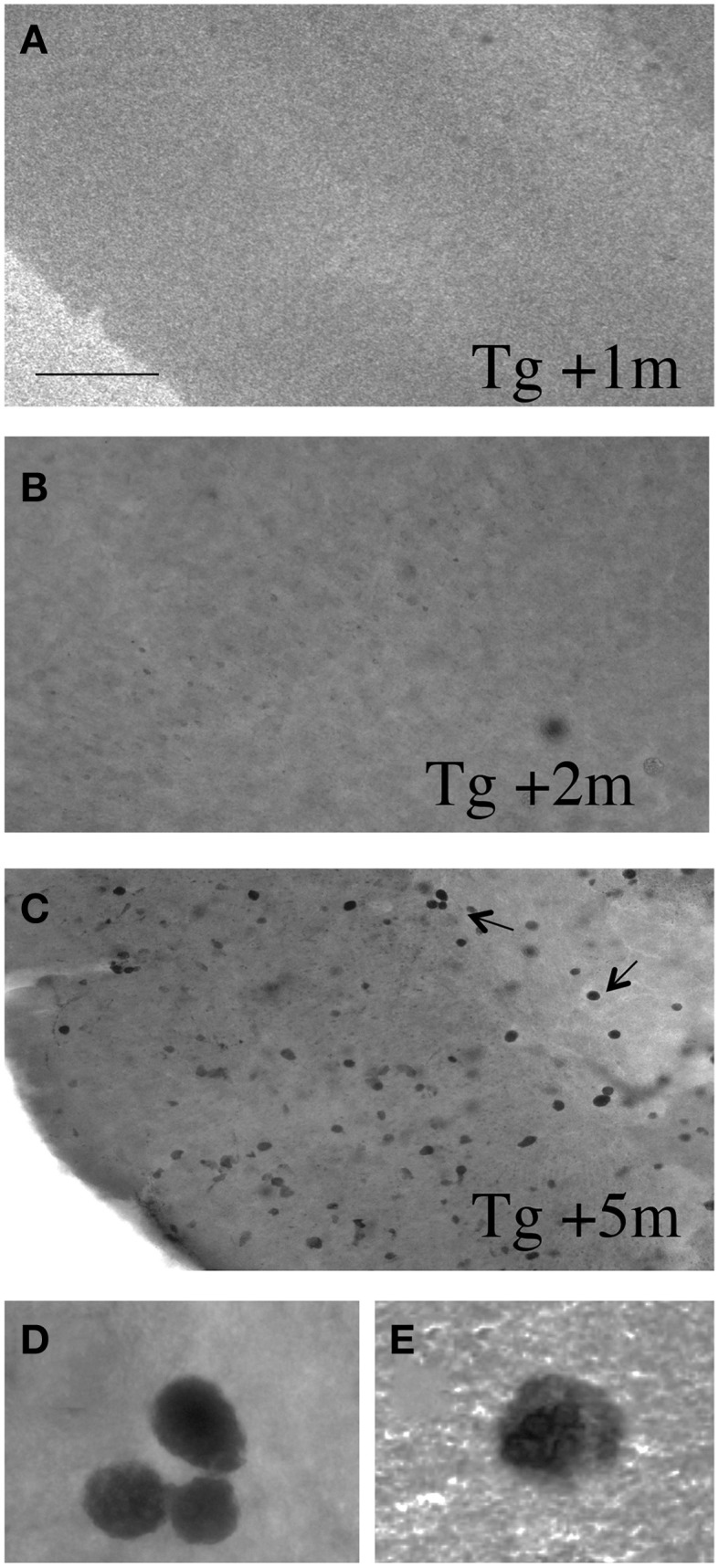
**Vibrosections from 4 month old APP_SDI mice (having no plaques at this stage) were incubated for 1 (A), 2 (B), or 5 (C–E) months, then fixed and stained for Aβ**. No staining was visible after 1 or 2 months of incubation, however, after 3 and more pronounced after 4 and 5 months several Aβ+ intracellular depositions were seen. The 2 pictures in the last row **(D,E)** show high magnifications taken from panel **(C)**. However, at any stage no extracellular plaques were found. Scale bar in *A* = 200 μm **(A–C)** and 12 μm **(D,E)**.

In order to study if slices develop plaques, vibrosections from 4 month old APP_SDI Tg mice (which display no plaques at this stage) were incubated for up to 5 months and then stained for Aβ plaques. Slices incubated for 1 or 2 months did not show any Aβ staining (Figures 3A,B). However, after 3 months and more pronounced after 4 and 5 months of incubation several intracellular Aβ-like depositions were seen, but no extracellular plaques were found (Figures 3C–E).

### Clearance of plaques

**Figure 4:**
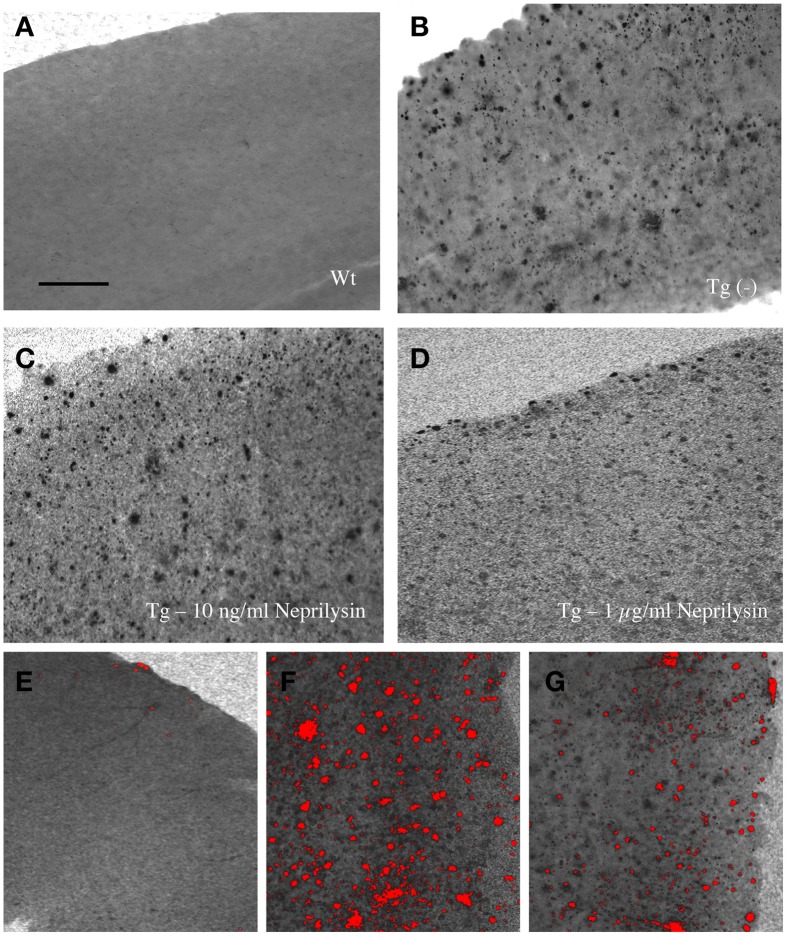
**Effects of neprilysin on beta-amyloid (Aβ) plaques in 9 month old amyloid-precursor protein_SDI mouse model vibrosections and computer-assisted analysis (E-G)**. Organotypic vibrosections of 9 month old wildtype (Wt, **A,E**) or transgenic (Tg, **B–D,F,G**) mice were prepared (120 μm thick) and cultured for 2 weeks without **(A,B)** or with 10 ng/ml **(C)** or 1 μg/ml **(D)** neprilysin, then fixed and immunohistochemically stained for beta-amyloid **(A–G)**. Note the decreased number of plaques in vibrosections incubated with 1 μg/ml recombinant neprilysin for 2 weeks **(D)**. The number of plaques was counted using computer-assisted imaging **(E–G)**, showing no plaques in Wt vibrosections **(E)**, a high number of plaques in Tg vibrosections **(F)** and a reduced number of plaques in the neprilysin treated vibrosections **(G)**. Scale bar in *A* = 200 μm **(A–G)**.

**Figure 5:**
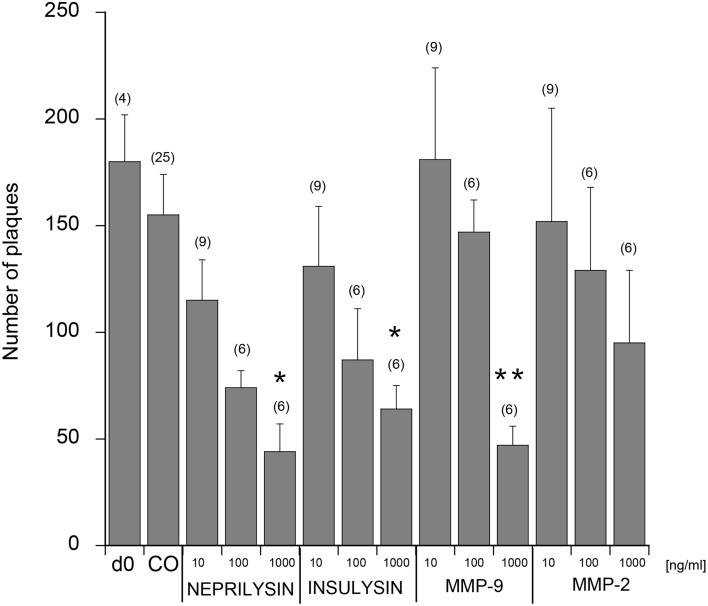
**Quantitative analysis of beta-amyloid (Aβ) plaques in the cortical vibrosections**. Vibrosections of 9 month old adult amyloid-precursor protein_SDI mouse model were cultured for 2 weeks without (CO) or with 10, 100, or 1000 ng/ml neprilysin, insulysin, matrix metalloproteinase (MMP) −2 or −9. Slices were then fixed and immunohistochemically stained for plaques with a beta-amyloid antibody. As a comparison the number of plaques is given from brain sections at day zero (d0). The number of beta-amyloid plaques was evaluated using computer-assisted microscopy. Values are given as mean ± SEM plaques per 2.5 mm^2^; the values in parenthesis give the number of analyzed slices. Statistical analysis was performed by a Kruskal–Wallis test and Dunn's *post-hoc* test (^*^p < 0.05; ^**^p < 0.01).

When 9 month old vibrosections of APP_SDI mice were cultured for 2 weeks with 1 μg/ml recombinant neprilysin (Figure 4D) or insulysin or MMP-9, a significant reduction of the number of plaques was observed (Figure 5) compared to controls (Figure 4A). The incubation with 1 μg/ml MMP-2 was not successful (Figure 5). No effect on plaque clearance was seen when the vibrosections were incubated with 100 or 10 ng/ml of all tested enzymes (Figures 4B,C, 5).

### Western blot analysis

**Figure 6:**
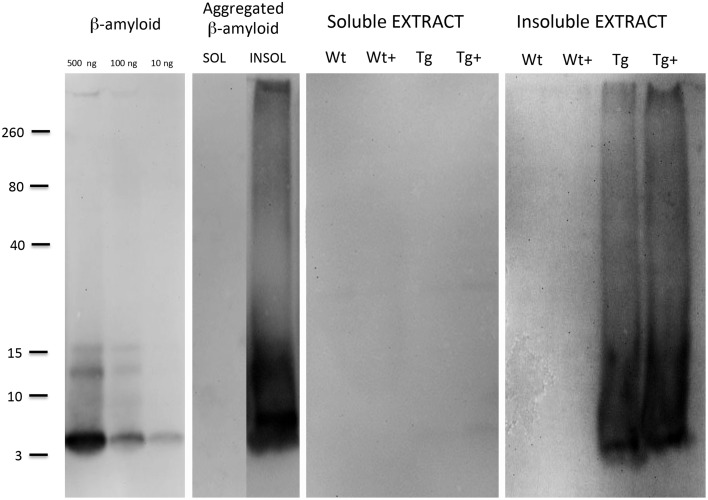
**Western Blot analysis of brain slice extracts**. Beta-amyloid (10–100–500 ng per lane), or aggregated beta-amyloid (2w 4°C + 2w 37°C) or slice extracts (either soluble or insoluble extracts) were loaded onto gels, separated for 25 min, blotted with 20% methanol and analyzed for beta-amyloid by enhanced chemiluminescence. Size markers are given on the left side as kDa. Slices were prepared from 9 month old wildtype (Wt) or APP_SDI transgenic (Tg) mice and incubated for 2 weeks with or without 1 μg/ml MMP-9 (Wt+ or Tg+).

The Aβ standards were detectable as a small peptide at approximately 4 kDa with a detection limit as low as 10 ng per lane (Figure 6). When aggregated Aβ was loaded several aggregated forms (mainly 8–12–16 kDa) were visible, however, also larger smear-like aggregates were found (Figure 6). No Aβ-immunoreactivity was seen in soluble aggregates or soluble slice extracts (Figure 6). When insoluble slice extracts were analyzed by Western Blot, no staining was seen in Wt mice, however, a strong staining was seen for 4–16 kDa Aβ forms and a strong larger smear (Figure 6). However, no clear difference was seen between MMP-9 treated or untreated vibrosections (Figure 6).

## Discussion

The organotypic brain slice and vibrosection model is well-established and serves as a validated tool to study neurodegeneration or neuroprotection (Stoppini et al., 1991; Gähwiler et al., 1997; Schatz et al., 1999; Weis et al., 2001; Humpel and Weis, 2002; Ullrich et al., 2011). Usually brain slices are derived from young postnatal brains, however, such slices are not useful, because plaques develop usually between 4 and 6 months of age in tg mice. Previously, we have developed a model from postnatal wildtype rats (Marksteiner and Humpel, 2008), however, such “Aβ-like depositions” are not fully comparable to the Aβ plaques-derived from adult brains. In the present study we developed for the first time an organotypic vibrosection model from adult AD mice, and demonstrate that this model shows Aβ plaques and is useful to screen for Aβ-degrading enzymes.

### Adult brain slices

Usually most brain slices are derived from postnatal animals, as it is difficult to properly prepare and maintain adult slices. Culturing of adult brain slices is very tricky and has been reported by only a few groups with several limitations, especially the short life time and reduced viability of the slices. Finley et al. (2004) cultured juvenile young adult (postnatal P20-21 days) hippocampal slices and reported maintained neuronal architecture and synaptic activity. Kim et al. (2013) established adult organotypic hippocampal slices in serum-free culture medium for 30 days and found well preserved astrocytes, neural processes and synapses. Su et al. (2011) cultured adult hippocampal slices which led to a time-dependent reproducible cell death. They reported that slices lost 50% of their cells after 6 days *in vitro*, while brain-derived neurotrophic factor supplementation promoted cell survival. Wilhelmi et al. (2002) reported the culturing of adult hippocampal slices in a cerebrospinal fluid-like medium, which markedly prolonged culture times up to 5–6 days. Anyhow, the need of special supplemented adult slice (serum-free) medium needs to be further optimized. So far, none of the published conditions for adult slices are accepted and established.

### Development of plaques in vibrosections

In our first approach we wanted to test if plaques develop in organotypic vibrosections. Thus, we cultured brain sections from 4 month old transgenic mice. At this stage no plaques are seen in the brain. We hypothesized that culturing of such slices for up to 5 months may induce Aβ plaque deposition, because 9 month old tg mice contain a large plaque load. Our data shows that up to 2 months of culturing, no Aβ-immunostaining was evident, however, after 3–5 months of culturing a strong intracellular Aβ immunoreactivity was found. At any stage we never observed extracellular Aβ plaque depositions in the vibrosections. Thus, this model may not be useful to screen for the effects of Aβ-degrading enzymes, but may rather be very useful to test the processes of Aβ processing, release and extracellular deposition to plaques. Thus, further experiments are necessary to test the conditions and factors, which may induce an extracellular plaque deposition *in vitro* similar as seen in the vivo situation.

### Plaques in advanced AD stages

In order to study plaques *in vitro* and to investigate Aβ-degrading enzymes, we developed an *in vitro* vibrosection model from 9 month old tg mice, which contain a high plaque load at this stage in the whole brain (Davis et al., 2004). Our data clearly show that culturing of 120 μm adult vibrosections for 2 weeks maintains many Aβ plaques. These plaques *in vitro* are typically of the same pattern and size as the plaques seen *in vivo*, showing strong Aβ depositions. The number of plaques did not increase during culturing for 2 weeks. Using Western blots we confirmed that the vibrosections contained aggregated insoluble Aβ at this stage. Further we show, that these plaques are surrounded by reactive healthy GFAP+ astrocytes. Similarly, also reactive Iba1+ microglia have been found in these slices. This is a typical cellular pattern, that reactive glial cells surround the plaques. However, we cannot exclude that additional traumatic effects at both sides of the vibrosection occurs due to slicing, which may influence the plaque composition.

### Degradation and clearance of plaques with neprilysin

Neprilysin is a 85–110 kDa plasma membrane glycoprotein of the neutral zinc metalloendo-peptidase family expressed by neurons and cerebrovascular smooth muscle cells. Neprilysin seems to be the most potent Aβ-degrading enzyme (Iwata et al., 2001) and several studies demonstrated its effect. In fact, several cleavage sites of NEP within the Aβ sequence have been identified (Grimm et al., 2013). However, it is worth mentioning that NEP cleaves monomeric Aβ_1–40_ more efficiently than Aβ_1–42_; *in vitro* NEP degraded 27% of monomeric Aβ_1–42_, but cleaved 73% of Aβ_1–40_ monomers (Grimm et al., 2013). It has been shown that intracerebral infusion of a recombinant soluble neprilysin from insect cells into AD mice improved memory and reduced Aβ accumulation in the brain (Park et al., 2013). Further an intravenous infusion of a NEP fusion protein resulted in dose-dependent clearance of Aβ, but did not affect brain Aβ, however, an intracranial infusion was successful (Walker et al., 2013). Liu et al. (2010) produced a secreted form of NEP by adeno-associated transduction and showed that plasma NEP decreased plasma Aβ by 30% and cleared brain Aβ by 28–55% within 3 months. In contrast, overexpression of NEP on the surface of leucocytes (by lentiviral induction) and periphal infusion reduced soluble Aβ in brain by 30% and lowered CAA Aβ by 50–60% (Guan et al., 2009). In contrast, another group fused NEP to albumin to increase plasma half-life time and showed that repated i.v. infusion for 4 months in Tg2576 mice (and rats and monkeys) cleared plasma Aβ but not brain or CSF Aβ (Henderson et al., 2014). Some studies were conducted also *in vitro*, however, their conclusions are limited due to the simplicity of the model. Miners et al. (2011) showed that NEP overexpression in primary human adult cerebrovascular smooth muscle cells counteracted Aβ-induced toxicity. In cultured astrocytes, ketamine (an NMDA antagonist) and MK-801 decreased NEP expression via de-phosphorylation of p38MAPK, suggesting that these signal pathways are linked to Aβ degradation (Yamamoto et al., 2013). All in all, the *in vivo* data are partly not conclusive, although NEP seems to have the most prominent Aβ-degrading activity. Our present data indeed show that recombinant NEP degrades Aβ plaques, but only at the highest concentration of 1 μg/ml. However, we cannot exclude that the added proteases underwent autodegradation or that the enzymes added to the medium did not efficiently diffuse into the slice. In this case it would be helpful to determine the catalytic activity in slice extracts under optimized conditions.

### Insulysin, MMPs, and other enzymes

Yan et al. (2006) reported that MMP-9 was very potent to degrade soluble fibrillar Aβ, but this ability was not shared by other enzymes examined, including ECE, IDE and neprilysin. In fact, MMP-9 was also the most potent enzyme to degrade Aβ in our hands. MMP-9 is a zinc dependent metalloprotease and initially synthesized as the inactive proenzyme pro-MMP-9, which is cleaved into an active form upon release by other proteases. MMP-9 is expressed in many cells in the brain, neurons, astroglia, microglia, vascular cells and is increased in the brain of AD patients (Dzwonek et al., 2004; Nalivaeva et al., 2008). MMP-9 is expressed by reactive astrocytes around Aβ plaques, and it is suggested that these cells may contribute in plaque clearance (Yan et al., 2006; Yin et al., 2006).

Our data show, however, in contrast to Yan et al. (2006) that also neprilysin and IDE was very potent to degrade Aβ plaques. This must not be necessarily a contrast, because, the cleavage site of the enzymes must be considered. Studies examining different fragments of Aβ report on the importance of 2 critical regions for aggregation at positions 17–21 and 30–35 (see Yan et al., 2006). The suggested cleavage site of MMP-9 is Phe20-Ala21 and Ala30-Ile31 (Yan et al., 2006). However, the plaques in the different AD mouse models are heterogenous, because the mutation sites differ within the Aβ sequence. In our APP_SDI mouse model 3 mutations have been inserted (Swedish K670N/M671L); Dutch E22Q (E693Q) and Iowa D23N (D694N). Thus, the effects of the different proteases can vary in the different AD mouse models used.

In addition, a number of enzymes or chemicals have been reported to degrade plaques, however, often their effects are not well characterized, regarding either dosage or specificity of the substrate or selectivity to find the targets. Such a new enzyme is e.g., nardilysin (a N-arginine dibasic convertase; NRDc, initially identified as a metalloendopeptidase of the M16 family), which has been reported to prevent amyloid plaque formation by enhancing alpha-secretase in an AD mouse model (Ohno et al., 2014). Finally, several other enzymes or substrates may induce Aβ degradation/clearance in the brain and may become a potent therapeutic target, such as e.g., BACE2, nattokinase or neprilysin-2 (Nalivaeva et al., 2008; Hsu et al., 2009; Hafez et al., 2010; Abdul-Hay et al., 2012). Further, different substrates (e.g., estrogens or BRI2 protein) may indirectly have Aβ-degrading activity via activation of Aβ-degrading enzymes (Kilger et al., 2011; Merlo and Sortino, 2012).

### Advantage of the adult AD *in vitro* model

Many of the reported enzymes have been shown to degrade soluble Aβ, but not all of them have been convincingly shown to degrade Aβ fibrils or compact plaques. The problems and limits are clearly that the effects of these enzymes cannot be easily studied *in vivo* in mouse models. The enzymes are large molecules, do not enter the BBB, have a wide range of substrates and side effects on peripheral organs or blood cells, when administered into the vein. Intraventricular infusions break up the BBB resulting in influx of blood-derived substrates and enzymes. Further, the stability of the applied enzymes is a problem, and only small amounts may enter the plaque site. An interesting experimental approach has been reported by Yan et al. (2006), who prepared 5 μm thin snap frozen cryostat sections from saline perfused AD mice (APP/PS1), incubated them in 70 nM protease (MMP-9, ECE, IDE, NEP) at 37°C for 5 days and stained them with Thioflavin S and observed degradation of plaques by MMP-9 only. This is an interesting model, however, since the slices have been fresh frozen, they are no longer considered as “living.” So far good *in vitro* models are missing and our novel model may provide for the first time a compact system to demonstrate clearance of plaques directly in a living mouse brain model allowing to study time- and dose-dependent effects.

### Limit of the model

This *in vitro* AD model has definitively several limitations. The most severe limitation is the low neuronal viability of the adult slices. Although it was out of focus to characterize the neuronal viability, we have preliminary data that the very sensitive dopaminergic and cholinergic neurons did not survive well in our adult wt or tg vibrosections even when incubated with growth factors. In fact, more work is needed to study and enhance the survival of neurons in adult slices, and the culture conditions must be adapted and optimized. Further, we cannot exclude that the enzymes (at highest concentration) may damage the vibrosections, especially the neurons. Cell death assays including e.g., propidiumiodine stainings, neuN immunostainings, and MAP-2 Western Blots may help to further characterize the adult slice model. So, far this present model may be limited but very powerful to study plaque development and degradation using Aβ-degrading enzymes.

Another problem, although not limited to the slice culture conditions, is the lack of Tau pathology, the second important pathological condition in AD. In most APP overexpressing models only Aβ plaques occur and no Tau pathology is evident. Alternatively, a triple transgenic mouse model (3xTg-AD, B6; 129-*P*sen1^tm1Mpm^Tg(APPSwe, tauB301L)1Lfa/J) could be useful which contains βA as well as Tau pathology. The problem is that the plaques and Tau pathology occur only at very lates age, approximately at 15–20 months, and it is not very convenient to use such old mice for cultures. Last not least the transgenic AD mouse model reflects more a genetic model of AD, which accounts for only <5% of all AD cases. As more than 95% are sporadic AD and the causes for AD are not known, animal models for sporadic AD have not been described to date.

### Clear or prevent?

The application of Aβ-degrading enzymes is a very interesting therapeutic option to degrade and clear plaques in severe AD patients. The problems of delivery, stability and selectivity must be resolved and thus this approach could become a potent technology to clear plaques at an end-stage. However, we also need therapeutic options to prevent plaques. Thus strategies need to be developed to apply such Aβ-degrading enzymes at a very early stage of the disease, such as in mild cognitive impairment. In fact, PET analysis shows that some individuals display already plaques at early age without having any cognitive symptoms. Such therapeutic approaches with proteases, however, must be safe, easy and specific. Organotypic vibrosections may help to understand and establish such therapeutic options with Aβ-degrading enzymes.

In conclusion our data provide for the first time a potent and powerful living brain vibrosection model containing a high number of plaques, which allows to rapidly and simply study the degradation and clearance of Aβ plaques *in vitro*.

## Author contributions

CH designed and analyzed the data and wrote the manuscript.

### Conflict of interest statement

The author declares that the research was conducted in the absence of any commercial or financial relationships that could be construed as a potential conflict of interest.

## References

[B1] Abdul-Hay S. O. Sahara T. McBride M. Kang D. Leissring M. A. (2012). Identification of BACE2 as an avid ß-amyloid-degrading protease. Mol. Neurodegener. 7:46. 10.1186/1750-1326-7-46 22986058 PMC3470943

[B2] Backstrom J. R. Lim G. P. Cullen M. J. Tökés Z. A. (1996). Matrix metalloproteinase-9 (MMP-9) is synthesized in neurons of the human hippocampus and is capable of degrading the amyloid-beta peptide (1-40). J. Neurosci. 16, 7910–7919. 8987819 10.1523/JNEUROSCI.16-24-07910.1996 PMC6579235

[B3] Bennett R. G. Duckworth W. C. Hamel F. G. (2000). Degradation of amylin by insulin-degrading enzyme. J. Biol. Chem. 275, 36621–36625. 10.1074/jbc.M006170200 10973971

[B4] D'Andrea M. R. Cole G. M. Ard M. D. (2004). The microglial phagocytic role with specific plaque types in the Alzheimer disease brain. Neurobiol. Aging 25, 675–683. 10.1016/j.neurobiolaging.2003.12.026 15172747

[B5] Davis J. Xu F. Deane R. Romanov G. Previti M. L. Zeigler K.. (2004). Early-onset and robust cerebral microvascular accumulation of beta-amyloid-protein in transgenic mice expressing low levels of a vasculotropic Dutch/Iowa mutant form of beta-amyloid-protein precursor. J. Biol. Chem. 279, 20296–20306. 10.1074/jbc.M312946200 14985348

[B6] Dzwonek J. Rylski M. Kaczmarek L. (2004). Matrix metalloproteinases and their endogenous inhibitors in neuronal physiology of the adult brain. FEBS Lett. 567, 129–135. 10.1016/j.febslet.2004.03.070 15165905

[B7] Eckman E. A. Reed D. K. Eckman C. B. (2001). Degradation of the Alzheimer's amyloid beta peptide by endothelin-converting enzyme. J. Biol. Chem. 276, 24540–24548. 10.1074/jbc.M007579200 11337485

[B8] Eckman E. A. Watson M. Marlow L. Sambamurti K. Eckman C. B. (2002). Alzheimer's disease beta-amyloid peptide is increased in mice deficient in endothelin-converting enzyme. J. Biol. Chem. 278, 2081–2084. 10.1074/jbc.C200642200 12464614

[B9] Farris W. Mansourian S. Chang Y. Lindsley L. Eckman E. A. Frosch M. P.. (2003). Insulin-degrading enzyme regulates the levels of insulin, amyloid beta-protein, and the beta-amyloid precursor protein intracellular domain *in vivo*. Proc. Natl. Acad. Sci. U.S.A. 100, 4162–4167. 10.1073/pnas.0230450100 12634421 PMC153065

[B10] Finley M. Fairman D. Liu D. Li P. Wood A. Cho S. (2004). Functional validation of adult hippocampal organotypic cultures as an *in vitro* model of brain injury. Brain Res. 1001, 125–132. 10.1016/j.brainres.2003.12.009 14972661

[B11] Gähwiler B. H. Capogna M. Debanne D. McKinney R. A. Thompson S. M. (1997). Organotypic slice cultures: a technique has come of age. Trends Neurosci. 20, 471–477. 10.1016/S0166-2236(97)01122-3 9347615

[B12] Glabe C. (2000). Does Alzheimer disease tilt the scales of amyloid degradation versus accumulation? Nat. Med. 6, 133–134. 10.1038/72215 10655093

[B13] Grimm M. O. Mett J. Stahlmann C. P. Haupenthal V. J. Zimmer V. C. Hartmann T. (2013). Neprilysin and Aβ clearance: impact of the APP intracellular domain in NEP regulation and implications in Alzheimer's disease. Front. Aging Neurosci. 5:98. 10.3389/fnagi.2013.00098 24391587 PMC3870290

[B14] Guan H. Liu Y. Police S. Kim M. H. Oddo S. LaFerla F. M.. (2009). Peripherally expressed neprilysin reduces brain amyloid burden: a novel approach for treating Alzheimer's disease. J. Neurosci. Res. 87, 1462–1473. 10.1002/jnr.21944 19021293 PMC2832596

[B15] Hafez D. Huang J. Y. Huynh A. M. Valtierra S. Rockenstein E. Bruno A. M.. (2010). Neprilysin-2 is an important β-amyloid degrading enzyme. Am. J. Pathol. 178, 306–312. 10.1016/j.ajpath.2010.11.012 21224067 PMC3069828

[B16] Henderson S. Andersson C. Janson J. Goldschmidt T. J. Appelkvist P. Bogstedt A.. (2014). Sustained peripheral depletion of amyloid-beta with a novel form of neprilysin does not affect central levels of amyloid-beta. Brain 137(Pt 2), 553–564. 10.1093/brain/awt308 24259408 PMC3914468

[B17] Hohsfield L. A. Ehrlich D. Humpel C. (2014). Intravenous infusion of NGF-secreting monocytes supports the survival of cholinergic neurons in the nucleus basalis of Meynert in hypercholesterolemia Brown Norway rats. J. Neurosci. Res. 92, 298–306. 10.1002/jnr.23309 24323796 PMC4311143

[B18] Hsu R. L. Lee K. T. Wang J. H. Lee L. Y. Chen R. P. (2009). Amyloid-degrading ability of nattokinase from *Bacillus subtilis* natto. J. Agric. Food Chem. 57, 503–508. 10.1021/jf803072r 19117402

[B19] Hu J. Igarashi A. Kamata M. Nakagawa H. (2001). Angiotensin-converting enzyme degrades Alzheimer amyloid beta-peptide: retards Amyloid beta aggregation, deposition, fibril formation; and inhibits cytotoxicity. J. Biol. Chem. 276, 47863–47868. 10.1074/jbc.M104068200 11604391

[B20] Humpel C. Weis C. (2002). Nerve growth factor and cholinergic CNS neurons studied in organotypic brain slices: implications in Alzheimer's disease? J. Neural Transm. Suppl. 62, 253–263. 10.1007/978-3-7091-6139-5_23 12456068

[B21] Iwata N. Iwata N. Tsubuki S. Takaki Y. Shirotani K. Lu B.. (2001). Metabolic regulation of brain Abeta by neprilysin. Science 292, 1550–1552. 10.1126/science.1059946 11375493

[B22] Kilger E. Buehler A. Woelfing H. Kumar S. Kaeser S. A. Nagarathinam A.. (2011). BRI2 protein regulates β-amyloid degradation by increasing levels of secreted insulin-degrading enzyme (IDE). J. Biol. Chem. 286, 37446–37457. 10.1074/jbc.M111.288373 21873424 PMC3199491

[B23] Kim H. Kim E. Park M. Lee E. Namkoong K. (2013). Organotypic hippocampal slice culture from the adult mouse brain: a versatile tool for translational neuropsychopharmacology. Prog. Neuropsychopharmacol. Biol. Psychiatry 41, 36–43. 10.1016/j.pnpbp.2012.11.004 23159795

[B24] Kurochkin I. V. (2001). Insulin-degrading enzyme: embarking on amyloid destruction. Trends Biochem. Sci. 26, 421–425. 10.1016/S0968-0004(01)01876-X 11440853

[B25] Lebson L. Nash K. Herber D. Carty N. Lee D. C. Li Q.. (2010). Trafficking CD11b-positive blood cells deliver therapeutic genes to the brain of amyloid-depositing transgenic mice. J. Neurosci. 30, 9651–9658. 10.1523/JNEUROSCI.0329-10.2010 20660248 PMC2929651

[B26] Ledesma M. D. Da Silva J. S. Crassaerts K. Delacourte A. De Strooper B. Dotti C. G. (2000). Brain plasmin enhances APP alpha-cleavage and Abeta degradation and is reduced in Alzheimer's disease brains. EMBO Rep. 1, 530–535. 10.1093/embo-reports/kvd107 11263499 PMC1083779

[B27] Lee C. Y. Landreth G. E. (2010). The role of microglia in amyloid clearance from the AD brain. J. Neural Transm. 117, 949–960. 10.1007/s00702-010-0433-4 20552234 PMC3653296

[B28] Leissring M. A. Farris W. Chang A. Y. Walsh D. M. Wu X. Sun X.. (2003). Enhanced proteolysis of beta-amyloid in APP transgenic mice prevents plaque formation, secondary pathology, and premature death. Neuron 40, 1087–1093. 10.1016/S0896-6273(03)00787-6 14687544

[B29] Liao M. C. Ahmed M. Smith S. O. Van Nostrand W. E. (2009). Degradation of amyloid beta protein by purified myelin basic protein. J. Biol. Chem. 284, 28917–28925. 10.1074/jbc.M109.050856 19692707 PMC2781437

[B30] Liu Y. Studzinski C. Murphy M. P. Klein R. L. Hersh L. B. (2010). Circulating neprilysin clears brain amyloid. Mol. Cell. Neurosci. 45, 101–107 10.1016/j.mcn.2010.05.014 20558294 PMC2923273

[B31] Marksteiner J. Humpel C. (2008). Beta-amyloid expression, release and extracellular deposition in aged rat brain slices. Mol. Psychiatry 13, 939–952. 10.1038/sj.mp.4002072 17712316

[B32] Marr R. A. Rockenstein E. Mukherjee A. Kindy M. S. Hersh L. B. Gage F. H.. (2003). Neprilysin gene transfer reduces human amyloid pathology in transgenic mice. J. Neurosci. 23, 1992–1996. 12657655 10.1523/JNEUROSCI.23-06-01992.2003 PMC6742010

[B33] Merlo S. Sortino M. A. (2012). Estrogen activates matrix metalloproteinases-2 and -9 to increase beta amyloid degradation. Mol. Cell. Neurosci. 49, 423–429. 10.1016/j.mcn.2012.02.005 22402435

[B34] Miners J. Kehoe P. Love S. (2011). Neprilysin protects against cerebral amyloid angiopathy and beta-amyloid-induced degeneration of cerebrovascular smooth muscle cells. Brain Pathol. 21, 594–605. 10.1111/j.1750-3639.2011.00486.x 21382117 PMC8094282

[B35] Nalivaeva N. N. Fisk L. R. Belyaev N. D. Turner A. J. (2008). Amyloid-degrading enzymes as therapeutic targets in Alzheimer's disease. Curr. Alzheimer Res. 5, 212–224. 10.2174/156720508783954785 18393806

[B36] Ohno M. Hiraoka Y. Nishi K. Saijo S. Matsuoka T. Tomimoto H.. (2014). Nardilysin prevents amyloid plaque formation by enhancing gamma-secretase activity in an Alzheimer's disease mouse model. Neurobiol. Aging 35, 213–222. 10.1016/j.neurobiolaging.2013.07.014 23954170

[B37] Park M. Lee J. K. Ahn J. Jin H. K. Park J. S. Bae J. S. (2013). Recombinant soluble neprilysin reduces amyloid-beta accumulation and improves memory impairment in Alzheimer's disease mice. Brain Res. 1529, 113–124. 10.1016/j.brainres.2013.05.045 23831521

[B38] Qiu W. Q. Walsh D. M. Ye Z. Vekrellis K. Zhang J. Podlisny M. B.. (1998). Insulin-degrading enzyme regulates extracellular levels of amyloid beta-protein by degradation. J. Biol. Chem. 273, 32730–32738. 10.1074/jbc.273.49.32730 9830016

[B39] Roher A. E. Kasunic T. C. Woods A. S. Cotter R. J. Ball M. J. Fridman R. (1994). Proteolysis of A beta peptide from Alzheimer disease brain by gelatinase A. Biochem. Biophys. Res. Commun. 205, 1755–1761. 10.1006/bbrc.1994.2872 7811262

[B40] Ryan D. Narrow W. C. F. H. Bowers W. J. (2010). An improved method for generating consistent soluble amyloid-beta oligomer preparations for *in vitro* neurotoxicity studies. J. Neurosci. Methods 190, 171–179. 10.1016/j.jneumeth.2010.05.001 20452375 PMC2902796

[B41] Schatz S. Kaufmann W. A. Saria A. Humpel C. (1999). Dopamine neurons in a simple GDNF-treated meso-striatal organotypic co-culture model. Exp. Brain Res. 127, 270–278. 10.1007/s002210050796 10452214

[B42] Selkoe D. J. (2002). Alzheimer's disease is a synaptic failure. Science 298, 789–791. 10.1126/science.1074069 12399581

[B43] Stoppini L. Buchs P. A. Muller D. (1991). A simple method for organotypic cultures of nervous tissue. J. Neurosci. Methods 37, 173–182. 10.1016/0165-0270(91)90128-M 1715499

[B44] Su T. Paradiso B. Long Y. S. Liao W. P. Simonato M. (2011). Evaluation of cell damagae in organotypic hippocampal slice culture from adult mouse: a potential model system to study neuroprotection. Brain Res. 1385, 68–76. 10.1016/j.brainres.2011.01.115 21303673

[B45] Tucker H. M. Kihiko M. Caldwell J. N. Wright S. Kawarabayashi T. Price D.. (2000). The plasmin system is induced by and degrades amyloid-beta aggregates. J. Neurosci. 20, 3937–3946. 10818128 10.1523/JNEUROSCI.20-11-03937.2000 PMC6772619

[B46] Ullrich C. Daschil N. Humpel C. (2011). Organotypic vibrosections: novel whole sagittal brain cultures. J. Neurosci. Methods 201, 131–141. 10.1016/j.jneumeth.2011.07.021 21835204 PMC3176904

[B47] Van Nostrand W. E. Porter M. (1999). Plasmin cleavage of the amyloid beta-protein: alteration of secondary structure and stimulation of tissue plasminogen activator activity. Biochemistry 38, 11570–11576. 10.1021/bi990610f 10471309

[B48] Walker J. Pacoma R. Ou W. Alves J. Mason D. E. Peters E. C.. (2013). Enhanced proteolytic clearance of plasma Amyloi-beta by peripherally administered neprilysin does not result in reduced levels of brain Amyloid-beta in mice. J. Neurosci. 33, 2457–2464. 10.1523/JNEUROSCI.3407-12.2013 23392674 PMC6619149

[B49] Wang D. S. Dickson D. W. Malter J. S. (2006). beta-Amyloid degradation and Alzheimer's disease. J. Biomed. Biotechnol. 2006:58406. 10.1155/JBB/2006/58406 17047308 PMC1559921

[B50] Weis C. Marksteiner J. Humpel C. (2001). Nerve growth factor and glial cell line-derived neurotrophic factor restore the cholinergic phenotype in organotypic brain slices of the basal nucleus of Meynert. Neuroscience 102, 129–138. 10.1016/S0306-4522(00)00452-8 11226676

[B51] Wilhelmi E. Schöder U. H. Benabdallah A. Sieg F. Breder J. Reymann K. G. (2002). Organotypic brain-slice cultures from adult rats: approaches for a prolonged culture time. Altern. Lab. Anim. 30, 275–283. 10.1371/journal.pone.0045017 12106005

[B52] Wisniewski H. M. Wen G. Y. Kim K. S. (1989). Comparison of four staining methods on the detection of neuritic plaques. Acta Neuropathol. 78, 22–27. 10.1007/BF00687398 2472039

[B53] Xie H. Guan J. Borrelli L. A. Xu J. Serrano-Pozo A. Bacskai B. J. (2013). Mitochondrial alterations near amyloid plaques in an Alzheimer's disease mouse model. J. Neurosci. 33, 17042–17051. 10.1523/JNEUROSCI.1836-13.2013 24155308 PMC3807029

[B54] Yamada T. Miyazaki K. Koshikawa N. Takahashi M. Akatsu H. Yamamoto T. (1995). Selective localization of gelatinase A, an enzyme degrading beta-amyloid protein, in white matter microglia and in Schwann cells. Acta Neuropathol. 89, 199–203. 10.1007/BF00309334 7538720

[B55] Yamamoto N. Arima H. Kasahara R. Taniura H. Hirate H. Sugiura T.. (2013). Ketamine reduces amyloid beta-protein degradation by suppressing neprilysin expression in primary cultured astrocytes. Neurosci. Lett. 545, 54–58. 10.1016/j.neulet.2013.04.016 23624023

[B56] Yan P. Hu X. Yin K. Bateman R. J. Cirrito J. R. Xiao Q.. (2006). Matrix metalloproteinase-9 degrades amyloid-beta fibrils *in vitro* and compact plaques *in situ*. J. Biol. Chem. 281, 24566–24574. 10.1074/jbc.M602440200 16787929

[B57] Yin K. J. Yan P. Hu X. Xiao Q. Pan X. Bateman R.. (2006). Matrix metalloproteinases expressed by astrocytes mediate extracellular amyloid-beta peptide catabolism. J. Neurosci. 26, 10939–10948. 10.1523/JNEUROSCI.2085-06.2006 17065436 PMC6674654

